# Does Additive Pressurized Carbon Dioxide Lavage Improve Cement Penetration and Bond Strength in Cemented Arthroplasty?

**DOI:** 10.3390/jcm10225361

**Published:** 2021-11-17

**Authors:** Kevin Knappe, Christian Stadler, Moritz M. Innmann, Mareike Schonhoff, Tobias Gotterbarm, Tobias Renkawitz, Sebastian Jaeger

**Affiliations:** 1 Department of Orthopedic Surgery, Heidelberg University, 69118 Heidelberg, Germany; moritz.innmann@med.uni-heidelberg.de (M.M.I.); tobias.renkawitz@med.uni-heidelberg.de (T.R.); 2 Laboratory of Biomechanics and Implant Research, Clinic for Orthopedics and Trauma Surgery, Heidelberg University, 69118 Heidelberg, Germany; Mareike.Schonhoff@med.uni-heidelberg.de (M.S.); sebastian.jaeger@med.uni-heidelberg.de (S.J.); 3 Department of Orthopedic and Trauma Surgery, Linz University, 4020 Linz, Austria; christian.stadler@kepleruniklinikum.at (C.S.); tobias.gotterbarm@kepleruniklinikum.at (T.G.)

**Keywords:** cemented arthroplasty, pressurized carbon dioxide lavage, high-pressure pulsatile saline lavage, cement penetration, aseptic loosening, revision arthroplasty

## Abstract

The modern cementing technique in cemented arthroplasty is a highly standardized and, therefore, safe procedure. Nevertheless, aseptic loosening is still the main reason for revision after cemented total knee or cemented total hip arthroplasty. To investigate whether an additional carbon dioxide lavage after a high-pressure pulsatile saline lavage has a positive effect on the bone–cement interface or cement penetration, we set up a standardized laboratory experiment with 28 human femoral heads. After a standardized cleaning procedure, the test implants were cemented onto the cancellous bone. Subsequently, the maximum failure load of the bone–cement interface was determined using a material testing machine to pull off the implant, and the cement penetration was determined using computed tomography. Neither the maximum failure load nor cement penetration into the cancellous bone revealed significant differences between the groups. In conclusion, according to our experiments, the additive use of the carbon dioxide lavage after the high-pressure pulsatile lavage has no additional benefit for the cleaning of the cancellous bone and, therefore, cannot be recommended without restrictions.

## 1. Introduction

During the last few decades, cementless fixation in total hip arthroplasty in Germany increased [1]. This is most likely the result of very good long-term outcomes [2,3,4]. Nevertheless, cemented endoprosthesis is still important in patients with osteoporosis, complex anatomy, hemiarthroplasty, or revision cases. In total knee arthroplasties, more than 90% are still implanted using bone cement. Excellent long-term results can be achieved using cemented implant fixation in total joint arthroplasty [5,6,7,8]. National registries demonstrate slight differences in the surgical technique for total knee arthroplasty. In the Netherlands, New Zealand, and Germany, 95% or more of the total knee arthroplasties (TKAs) are performed using the cemented technique [9,10,11] compared with only 92% in Sweden in 2019 [12]. In total hip arthroplasty (THA), those differences are even more obvious. In Sweden, a total of 76% of THAs are cemented, whereas, in Denmark, only 29% are implanted in this way [13]. Even though there are regional differences, the implantation procedures and technique are standardized. High-pressure pulsatile saline lavage irrigation, drying the bone bed, drilling holes in the tibia, and applying vacuum-mixed-cement to the implant and bone are mandatory steps in the implanting of TKAs [14]. The third generation cementing technique is performed in THA. The necessary steps are: a properly prepared bone bed through aggressive rasping, using high-pressure pulsatile saline lavage irrigation, using a distal cement restrictor, applying vacuum-mixed-cement in a retrograde technique into the femur via a cement gun, pressurizing the cement, and inserting the stem with a distal centralizer [15]. However, even though the cementing technique has evolved over the decades, aseptic loosening is still the main reason for the revision after the cemented TKA and THA [9,16,17].

The adequate cleaning of the cancellous bone prior to the cement application increases the implant stability. In bone with reduced bone density, a pulsed lavage can have a protective effect on the implant stability [18]. The integrity of the bone–cement interface can even be reduced by the presence of blood between the bone and cement [19]. Knowing this, the removal of bone marrow containing residual bone material, blood, and fat is obligatory. The depth of penetration of the cement and bone–cement interface is directly related to prior cleaning. Furthermore, 3–5 mm is the desired cement thickness for stable anchorage [20,21]. Not only does polymerizing cement not have the harmful thermal effects described for a cement thickness of more than 5 mm [21,22]; more importantly, the implant failure rates are significantly lower compared with a cement thickness of less than 2 mm [23]. In THA, the use of a high-pressure pulsatile lavage increased from 13% to 78% between 1998 and 2010 in Germany [24]. This shows that using a high-pressure pulsatile lavage quickly established itself as a standard application in the field of cemented arthroplasty.

A well observed and described procedure of cleaning the cancellous bone is using a high-pressure pulsatile lavage and drying the bone bed with suction and an abdominal cloth. However, fat or blood may remain, which prevents the cement from penetrating the cancellous bone. Using a pulsatile lavage has been shown to be more efficient than manual rinse cleaning by a bladder syringe alone [25,26,27,28,29]. Using a high-pressure pulsatile lavage before cementing showed less radiolucent lines in TKA compared to the reference group [30]. This could also be shown in unicompartmental knee arthroplasty (UKA), where the pulsed lavage group only showed radiolucent lines in 4% of all four of the zones compared to the reference group with 22% [25]. A high-pressure carbon dioxide lavage is available for better cleaning of the cancellous bone and simultaneous drying of the bone [31]. It is recommended to be additionally used after conducting a high-pressure pulsatile saline lavage. In 2019, Gapinski et al. used sterile compressed CO_2_ in addition to a pulsatile lavage in 303 total knee endoprostheses. According to the Knee Society Radiographic Evaluation System, a significant deeper cleaning could be shown in three of the seven zones [32]. A significant deeper bone cement penetration could be shown in a cadaveric study using a combination of pressurized carbon dioxide and common pulsatile saline lavage [33]. In addition, in an experimental study using human radii, the bone–cement interface was stronger when the syringe lavage was compared to the carbon dioxide lavage [34]. In a standardized laboratory setup using a standardized carbon foam model, a deeper cleaning by using a compressed carbon dioxide lavage in addition to a saline high-pressure pulsatile lavage was shown. Nevertheless, the effect was not significant [35]. In contrast, embolic events were reported using a carbon dioxide lavage in intramedullary cleaning during cemented THA [36,37].

The aim of this experimental cadaver study was to investigate two different methods of bone cleaning and their effect on cement penetration and bone–cement interface stability. For this purpose, a conventional pulsatile lavage was compared with a combination of a pulsatile lavage and an additional compressed carbon dioxide lavage.

## 2. Materials and Methods

The study was divided into two separate investigations. The cement penetration was analyzed in the first part, and the bone–cement interface stability was analyzed in the second part. The study was conducted according to the guidelines of the Declaration of Helsinki and approved by the local ethics committee.

### 2.1. Test Setup and Preparations

**Figure 1:**
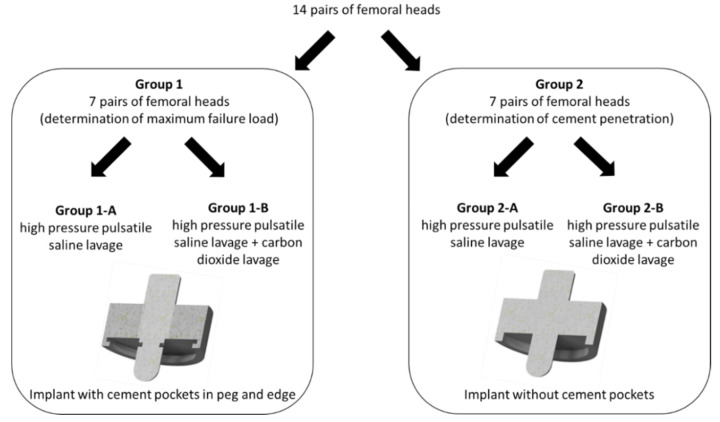
Distribution of the 28 femoral heads to the different cleaning procedures and different test implants for either determination of maximum failure load or cement penetration.

We investigated implant stability and cement penetration in 14 pairs of fresh frozen human femoral heads (28 in total). Femoral heads provided sufficient cancellous bone and made the test setup independent of different implant designs in TKA and THA. Seven pairs each were randomly assigned to Groups 1 (maximum failure load) and 2 (cement penetration). The specimens were randomly allocated with regard to right and left hip to Group A (high-pressure pulsatile lavage) or Group B (high-pressure pulsatile lavage with additional carbon dioxide lavage) by means of a computer-generated list (Randlist 1.2; Datinf GmbH, Tübingen, Germany) (Figure 1). In Group 1, the mean donor data showed an age of 74 ± 12.5 years, a height of 179 ± 5.9 cm, a weight of 107.3 ± 22.1 kg, and a body mass index of 33.2 ± 5.6 kg/m^2^. All donors in Group 1 were male. In Group 2, the mean donor data showed an age of 66.6 ± 18.3 years, a height of 170 ± 9.6 cm, a weight of 92.2 ± 37.2 kg, and a body mass index of 31.2 ± 11.4 kg/m^2^. Four of the donors in Group 2 were male and three were female. The bone mineral density was evaluated using standard hip parameters for dual-energy X-ray absorption (DXA) (Hologic QDR-2000, Marlborough, MA, USA). This allowed us to compare the groups and to assess bone quality.

**Figure 2:**
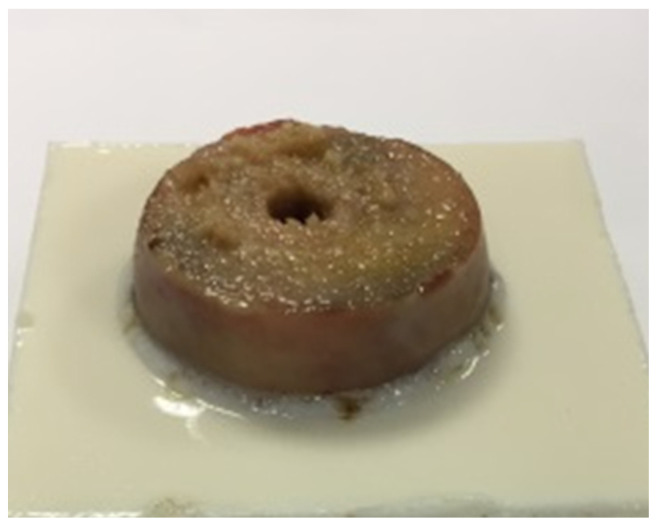
Embedded femoral head with drilled hole for the peg, prior to cleaning procedure.

**Figure 3:**
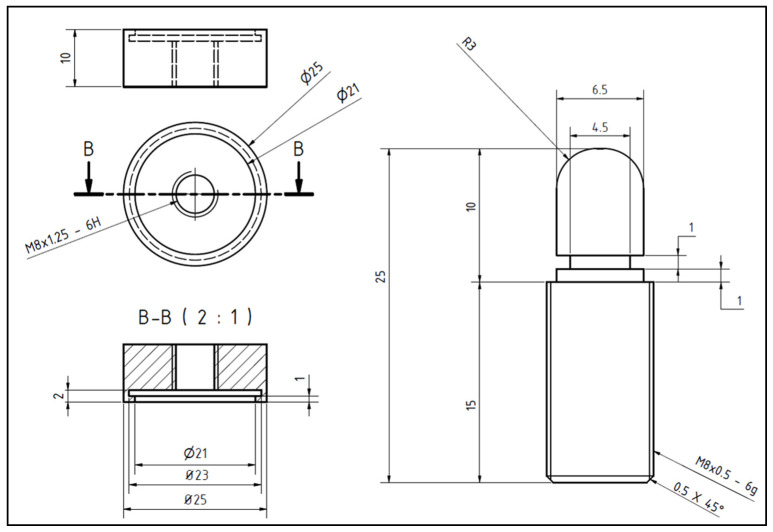
Technical drawing in mm of the test implant that was used in Group 1 (with undercuts/ cement pocket).

The specimens were casted in a mold using synthetic resin (Rencast FC 53, Huntsman Advanced Materials GmbH, Bergkamen, Germany) in order to be able to integrate them into the testing setup. All femoral heads were trimmed slightly above the equator to remove the fovea capitis femoris and to preserve the maximum volume of cancellous bone (Figure 2). In the right–left comparison, the identical resection heights were selected. By measuring the sliced fragments, the adherence to the resection heights was checked again. Since the cement–bone interface was investigated in this experimental study, we manufactured aluminum test implants as part of the test standardization following the peg design of the femoral component in TKA (Figure 3). In addition, a central hole for the peg with a diameter of 9 mm and a depth of 9 mm was drilled in the center of the fixed and resected femoral head (Figure 2). The hole diameter in relation to the peg diameter was chosen to allow a homogeneous cement mantle and to avoid a press fit.

### 2.2. Cancellous Bone Cleaning

The paired heads were divided into Group 1 (determination of the maximum failure load) and Group 2 (determination of cement penetration). This resulted in Group 1-A and Group 1-B to determine the maximum failure load and in Group 2-A and 2-B to determine the cement penetration of the different cancellous bone cleaning techniques, as described in the following section (Figure 1).

**Figure 4:**
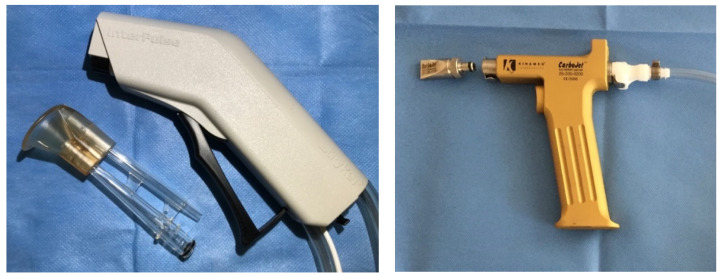
Pulsatile high-pressure saline lavage InterPulse with its bone cleaning tip (**left**) and carbon dioxide lavage CarboJet with its wide-angle-knee-nozzle (**right**).

In Group 1-A and 2-A, femoral heads were cleaned by high-pressure pulsatile saline lavage and dried by a compress. High-pressure pulsatile saline lavage was performed using the InterPulse with its bone cleaning tip (REF 0210-010-00; Stryker, Kalamazoo, MI, USA) (Figure 4 left). A flushing volume of one liter was used. The distance to the surface was determined by the splash-shield of the bone cleaning tip. While flushing, the lavage was continuously moved over the surface of the specimen.

In Group 1-B and 2-B, after cleaning the cancellous bone by high-pressure pulsatile saline lavage, as done in Group 1-A and 2-A, pressurized carbon dioxide lavage was performed additionally using the device CarboJet (Kinamed, Camarillo, CA, USA) with the supplied wide-angle-knee-nozzle (Figure 4 right) to dry the bone and to test for additional cleaning effects. According to manufacturer’s information, we performed this procedure for exactly 30 s with the nozzle slightly touching the cancellous bone in an angle of 45° to the plan surface of the cancellous bone.

### 2.3. Cementing Technique and Implantation of Test Implants

**Figure 5:**
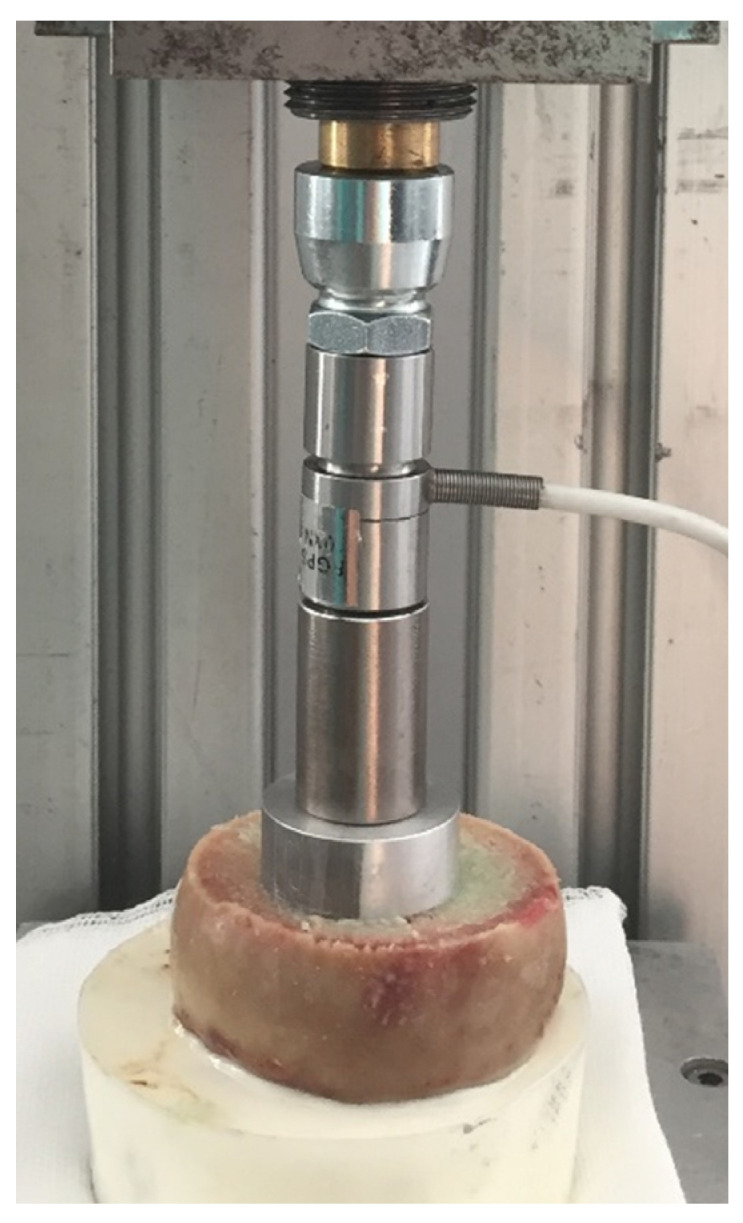
Femoral head (**bottom**) with round test implant cemented to the bone surface and the force measurement system (**top**).

Immediately after cleaning and drying of the cancellous bone, the cementation process was started. The bone cement (PALACOS R+G, Heraeus, Hanau, Germany) was mixed under vacuum (Optivac^®^, Zimmer Biomet Holdings, Warsaw, IN, USA) and applied with a cement gun. Early cement timing for vacuum mixed cement at a room temperature of 25 ± 1 °C was used. We applied the still sticky bone cement to the undersurface of the implant 50 s after the start of the mixing process. In the next step, the cement was applied to the bone at 90 s using the cement gun. The impaction of the implant was performed 150 s after the start of the mixing process. After impaction with manual compression, the ligament tension force was applied 180 s after the start of the mixing by a clamping system with fine thread (Figure 5). The ligament tension force was 180 N, as reported by Clarius et al., for unicompartmental knee arthroplasty [38,39]. The applied force was controlled during cement curing by means of a force measurement system. After reaching the end of the final curing phase (10 min after start of mixing), the ligament tension force was removed. The described procedure of cementation was performed identically in all used specimens regardless of which group they belonged to.

### 2.4. Determination of Maximum Failure Load

**Figure 6:**
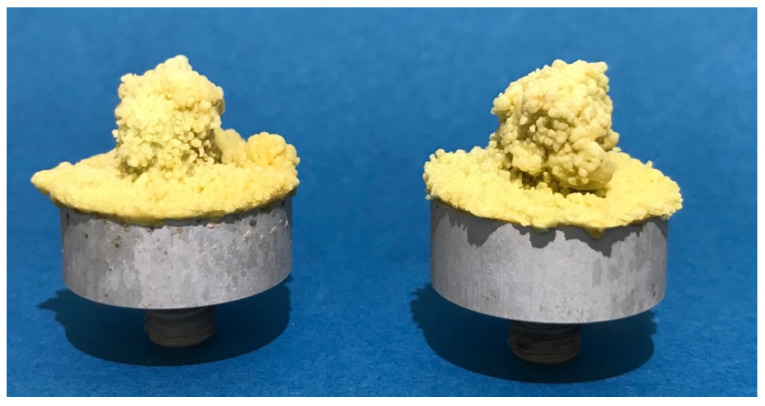
Already implanted test implants with cement after removal of all organic components.

In accordance with ISO 5833, the maximum load to failure of the bone–cement interface was determined 24 ± 2 h after cementation using a pull-out test. The specimens were stored in a refrigerator at +4 °C and kept at room temperature for 2 h before the start of the experiment. The test was performed for Group 1-A and 1-B using a material testing machine (Zwick Roell, Z005, Ulm, Germany) under displacement control. The implant was pulled out at a traverse speed of 2 mm/min. The resulting force was recorded, and the maximum force values were defined as failure load (Figure 6).

### 2.5. Cement Analysis/Cement Penetration

**Figure 7:**
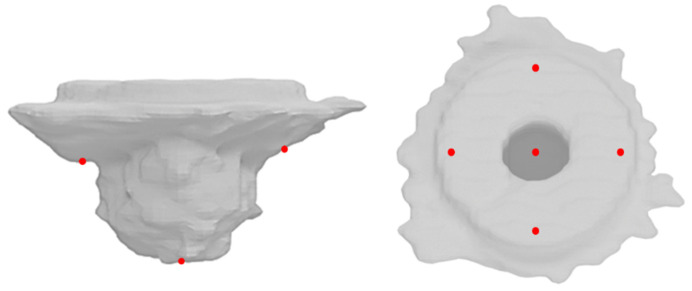
Defined measurement points to determine the cement penetration in Group 2-A and 2-B.

For analyzing cement penetration, we used an implant design without undercuts (Figure 1, bottom right). In addition, we coated the Group 2-A and 2-B implants with a wax-based release agent spray (goessl pfaff^®^, Karlskron, Germany). This enabled us to remove the implant after complete hardening of the bone cement without causing defects to the cement mantle. Subsequently, we obtained CT images (SOMATOM Emotion, Siemens Healthcare GmbH, Erlangen, Germany) of all 14 femoral heads of Group 2-A and 2-B with a slice thickness of 0.75 mm without metal artifacts. In the first step, the cement was segmented from the CT scans using the ITK-SNAP software [40]. Then, the cement volume and cement penetration were analyzed with the Geomagic Studio Program (Raindrop Geomagic, NC, USA). In addition, with regard to cement penetration depth, four different points in the plane and two further points under the peg were measured. Only the cement that penetrated the bone was analyzed (Figure 7). The cement outside the test implants and on top of the surface was not included in the calculation as it did not penetrate the cancellous structure.

### 2.6. Statistics

Prior to the start of the experimental study, a sample size calculation was performed using G*Power 3.1 (Kiel University, Kiel, Germany) [41]. This was based on the reported data by Kalteis et al. [29]. Input parameters to compute the required sample size were tails: two, effect size d: 2.031, α err prob: 0.05, and power (1-β err prob): 0.95. This results in the output parameters sample size 6 for each group and an actual power of 0.97. The data were evaluated descriptively using the arithmetic mean standard deviation, minimum, and maximum. Pre-analysis, the normal distribution of the data was evaluated using a Shapiro–Wilk-test, and the homogeneity of variance was verified using the Levene test. We conducted a two tailed *t*-test for independent samples to assess effects between the two groups on the parameters of bone mineral density, maximum failure load, cement penetration, and cement volume. All data were analyzed using SPSS V.25 (IBM Corp. Released 2017. IBM SPSS Statistics for Windows, Version 25.0. IBM Corp., Armonk, NY, USA) with a significance level of *p* < 0.05.

## 3. Results

### 3.1. Bone Mineral Density

The *t*-test revealed no statistically significant difference in the bone mineral density between Group 1-A 0.758 ± 0.10 g/cm^2^ and Group 1-B 0.751 ± 0.12 g/cm^2^, t(6) = 0.467, *p* = 0.657, (95% CI [−0.028, 0.041]). It also showed no statistically significant difference between Group 2-A 0.998 ± 0.21 g/cm^2^ and Group 2-B 0.976 ± 0.24 g/cm^2^, t(5) = −1.575, *p* = 0.176, (95% CI [−0.093, 0.022]).

### 3.2. Group 1-Bond Strength of the Bone–Cement Interface

**Figure 8:**
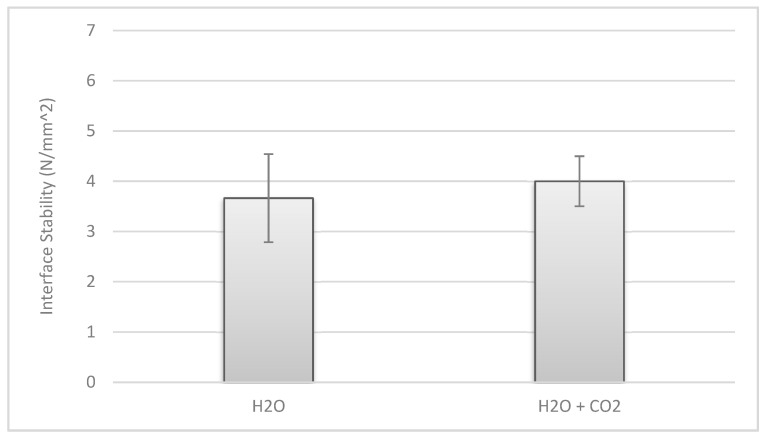
Force needed to pull off test implants (Group 1-A (**left**); 1-B (**right**)).

The *t*-test revealed no statistically significant difference for the additional use of a carbon dioxide lavage prior to cement application. The needed force to pull off the implant was 3.7 ± 0.9 N/mm^2^ in Group 1-A (saline lavage only) and 4.0 ± 0.5 N/mm^2^ in Group 1-B (saline lavage + carbon dioxide lavage). This difference of 0.3 N/mm^2^ showed no significance −t(6) = −1.029, *p* = 0.343 (95% CI [−1.206, 0.492]) (Figure 8).

### 3.3. Group 2-Cement Penetration

**Figure 9:**
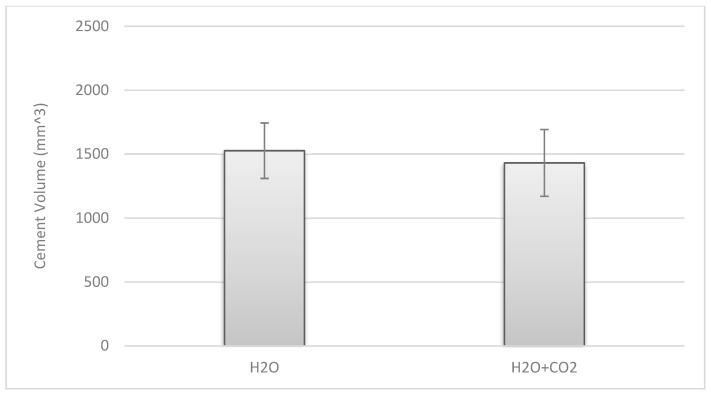
Cement volume penetrating the cancellous bone (Group 2-A (**left**); 2-B (**right**)).

The *t*-test also revealed no statistically significant difference for the additional use of a carbon dioxide lavage prior to cement application in terms of cement penetration. In Group 2-A, in which only a saline high-pressure lavage was used, the mean cement volume that penetrated into the cancellous bone was 1526.5 ± 217 mm^3^ according to the 3D analysis via itk-Snap [40]. In Group 2-B, where an additional carbon dioxide lavage was used, the mean cement volume was 1431.1 ± 963 mm^3^. The difference of 95.4 mm^3^ was not significant −t(6) = 1.815, *p* = 0.119 (95% CI [−33.19, 223.94]) (Figure 9).

**Figure 10:**
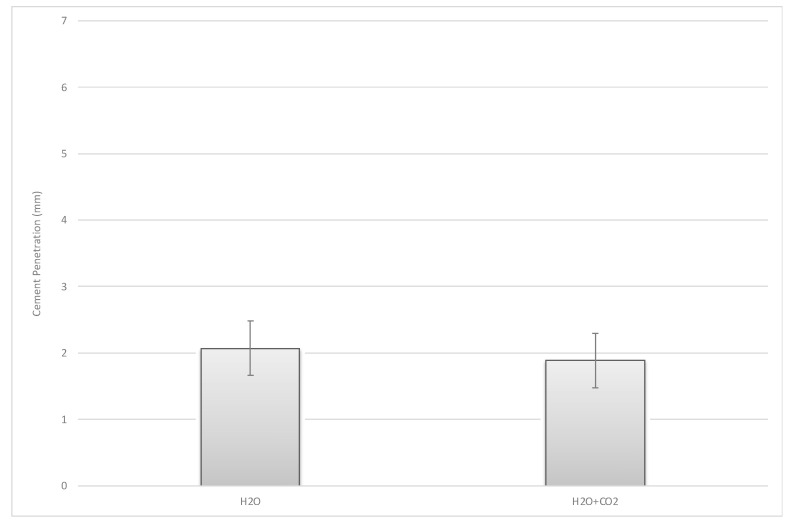
Mean cement penetration measurement point in the plane of cementation.

**Figure 11:**
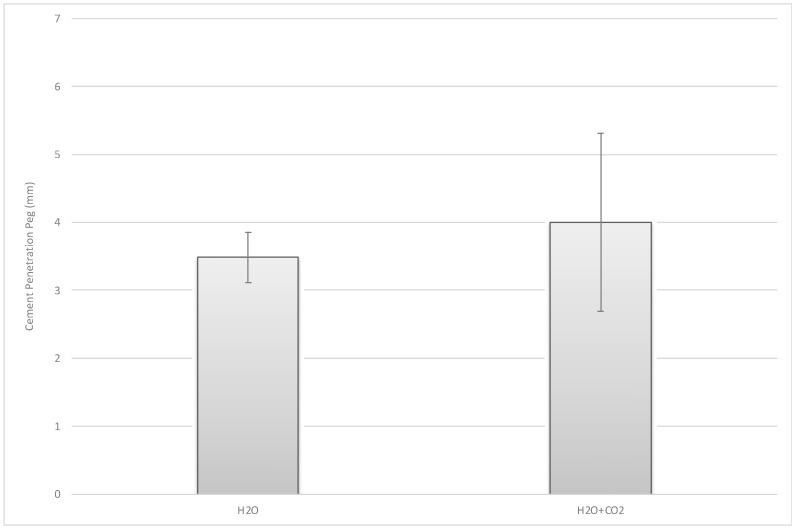
Mean cement penetration measurement points beneath the peg.

No significant difference could be found with regard to the depth measurement carried out at all six representative locations (four measurement points in the plane area t(6) = 0.91, *p* = 0.40 (95% CI [−0.314, 0.685]; two measurement points beneath the peg t(6) = −1.04, *p* = 0.34 (95% CI [−1.729, 0.700]) (Figure 7, Figure 10 and Figure 11).

## 4. Discussion

Since cementless THA has very good long-term results, the number of non-cemented total joint replacements rose during the last few decades [1,2,3,4]. In Germany, for example, 77.6% of the primary THAs were implanted without cement in 2020 [42]. Nevertheless, cemented endoprosthesis is still important in patients with osteoporosis, complex anatomy, hemiarthroplasty, or revision cases. In Germany in 2020, less than 2% of all the TKAs were implanted using no bone cement [42]. During the last three decades, cemented arthroplasty proved to be an excellent procedure in total joint replacement. Additionally, even though there are regional differences in whether a cemented or non-cemented technique is performed, a worldwide state of the art cementing technique has been established [14,15]. The main part of the third generation cementing technique is the adequate cleaning of the cancellous bone via a pulsatile saline high-pressure lavage. This crucial step in the process of cementing improves the interdigitation and sustainability of the bone–cement interface [18]. In a standardized laboratory setup, we compared two different lavage procedures in 28 paired human femoral heads. Half of them were cleaned by a high-pressure pulsatile lavage and a compress. For the other half, a high-pressure pulsatile lavage and additional carbon dioxide lavage was used. In two separate experiments, we tested for the bond-strength of the bone–cement interface and cement penetration in the cancellous bone. Our data showed no significant difference between the different cleaning procedures. Since even the presence of blood between the bone and cement can reduce the integrity of the bone–cement interface [19], we would have expected higher bond-strength in the group that has been edited additionally by a carbon dioxide lavage (Group 1-B) and not only by a compress (Group 1-A). In addition, knowing that cleaning is directly related to the cement penetration into the cancellous bone, we would have also expected higher volumes of cement penetration in the carbon dioxide cleaned group (Group 2-B), but there was also no significant difference to the group that had been dried only by a compress (Group 2-A). In our standardized laboratory setup, we used frozen human femoral heads, which, after defrosting, contained the same bone marrow and blood as live bones. Since a carbon dioxide lavage is recommended to be used after a saline pulsatile lavage, our data suggest that this procedure is not better than using a compress. Perhaps, fluids that remain in the cancellous bone after the lavage are not that obstructive for bone cement to penetrate into the bone [19]. It is even possible that the compress dried as much or more of the flushing medium than the carbon dioxide lavage was able to do. An experimental study using eight human radii showed that, in using a syringed saline or carbon dioxide lavage cleaning, there was a significantly stronger bone–cement interface in the four radii that were prepared by carbon dioxide lavage alone [34]. Since this study was not performed according to the manufacturer’s information in which a carbon dioxide lavage is recommended to be added to the high-pressure pulsatile lavage and a relatively low case number was used, the obtained data of this experiment cannot be applied to our study.

In addition, our data suggest that a carbon dioxide lavage had no additional cleaning effect, i.e., no bone marrow or remnants were harvested that had not been purified by a saline lavage. Knowing that using a system that requires additional devices and materials always comes at a financial cost, it seems debatable. Even additional waste must be considered if orthopedic surgery is to be more sustainable [43,44,45]. The usage of an additional device also takes time. Additional operation time is related to a higher possibility of infections and postoperative complications [46,47,48,49,50]. Therefore, an extensive duration of surgeries has to be avoided if possible. Since our greatest good is patient safety and knowing about reported cases of embolisms using a carbon dioxide lavage, the added value of this procedure has to be carefully tested [36,37]. Using a simplified cadaver laboratory setup, it is uncertain how the different procedures perform in humans if used in different anatomical regions in cemented arthroplasty. We used the cancellous structure of paired femoral heads to investigate the bone–cement interface and to determine the cement penetration. The orientation of the trabeculae differs from other regions. However, the paired femoral heads allowed us a high degree of standardization. Stability in the cemented joint replacement is achieved via the interdigitation of the bone cement into the cancellous bone. Since this is in contrast with uncemented arthroplasty, in which stability is gained through a process of growing bone to the prosthesis called osteoconduction [51,52,53,54], this should not be a significant factor.

In conclusion, the additive use of a carbon dioxide lavage after a high-pressure pulsatile saline lavage has no additional benefit in cleaning the cancellous bone in a laboratory setup and, therefore, cannot be recommended without restrictions.

## Data Availability

The data presented in this study are available within this article.
